# Short report: Associations of family characteristics and clinicians’ use of caregiver coaching in early intervention

**DOI:** 10.1177/13623613251317780

**Published:** 2025-02-11

**Authors:** Alyssa M Hernandez, Diondra Straiton, David S Mandell, Brooke Ingersoll, Samantha Crabbe, Sarah Rieth, Melanie Pellecchia

**Affiliations:** 1 Department of Psychology, University of Pennsylvania, USA; 2 Department of Psychiatry, University of Pennsylvania, USA; 3 Department of Psychology, Michigan State University, USA; 4 Department of Child and Family Development, San Diego State University, USA

**Keywords:** autism services, caregiver-mediated intervention, community-based implementation, early intervention, family characteristics

## Abstract

There is a great demand for quality early intervention services for autistic children and their families. Caregiver-mediated interventions are critical components of evidence-based early intervention. However, their implementation in publicly funded systems is often done with poor fidelity. Qualitative evidence suggests that family characteristics impact clinicians’ use of caregiver-mediated intervention coaching strategies. We estimated associations between family characteristics and clinicians’ use of a caregiver-mediated intervention in a publicly funded early intervention system, leveraging data from a pilot randomized trial. Data were collected from 12 clinicians and 34 families. We used multiple linear regression models to estimate associations. The association between household income and clinicians’ overall coaching fidelity demonstrated a quadratic trend (*b* *=* −10.4, standard error = 4.1, *p* < 0.05) with low fidelity for low- and high-income families relative to middle-income families. Use of in vivo feedback, one component of coaching, was similarly associated with income (*b* *=* −0.5, standard error = 0.2, *p* < 0.05). Clinicians’ coaching fidelity was lower among families in exclusively English-speaking homes than for families who speak other languages (*b* = 18.4, standard error = 8.8, *p* < 0.05). Results suggest that associations between family characteristics and clinician behavior may be more nuanced than previously understood.

**Lay abstract:**

There is a high demand for quality early intervention services for autistic children and their families. A key part of effective early intervention is teaching caregivers how to support their child’s development through caregiver-mediated interventions. However, in publicly funded programs, these strategies are often not followed correctly. Some studies suggest that family characteristics may influence how well clinicians apply these coaching methods. In this study, we explored the connection between family factors, like household income and language spoken at home, and the way clinicians coached families. We found that clinicians used coaching strategies less consistently with both lower- and higher-income families compared to middle-income ones. In addition, families that spoke only English at home received less consistent coaching than those who spoke other languages. These findings highlight the complex relationship between family background and how early intervention services are delivered, suggesting a need for further research.

Early intervention (EI) services are critical to enhance autistic children’s outcomes (Fuller & Kaiser, 2020; Reichow & Wolery, 2009; Zwaigenbaum et al., 2015). Federal policy mandates that EI help caregivers support their child’s development (Individuals With Disabilities Education Act (IDEA), 2004). Caregiver-mediated interventions (CMIs) involve clinicians coaching caregivers to use strategies that foster children’s development. CMI improves children’s social skills and communication, and reduces disruptive behavior (Cheng et al., 2023; Hume et al., 2021).

In community-based studies of CMI, outcomes tend to be worse than in university-based studies (Brookman-Frazee et al., 2012; Green et al., 2022), at least in part because community clinicians are less likely to coach caregivers (Aranbarri et al., 2021; Pellecchia et al., 2022). When clinicians coach caregivers in the community, it is often with lower fidelity than observed in university-based studies (Pellecchia et al., 2022). Clinicians vary in their intentions to implement specific coaching components (Lawson et al., 2022; Pellecchia et al., 2024), with some used more than others (Pellecchia et al., 2023b).

Racially minoritized and socioeconomically disadvantaged autistic children have less access to and receive lower-quality behavioral healthcare than their White (Begeer et al., 2009; Liu et al., 2023; Magaña et al., 2012, 2013) and socioeconomically advantaged peers (Carr et al., 2016; Fountain et al., 2012). This may be in part due to clinicians’ perceptions, as well as the experienced barriers related to family characteristics and living conditions, that hinder CMI coaching (Straiton et al., 2021; Tomczuk et al., 2022).

The present observational study leverages data from a community-based trial of a CMI (*Project ImPACT*). We examined whether family characteristics were associated with clinicians’ use of caregiver coaching.

## Methods

This study comprised a secondary analysis of data collected from a pilot randomized trial of *Project ImPACT*, a caregiver-mediated Naturalistic Developmental Behavioral Intervention (NDBI; Ingersoll & Dvortcsak, 2019). The parent study was approved by the City of Philadelphia’s Institutional Review Board (IRB) and involved the collaboration of EI agency partners. The trial design and results are published elsewhere (Pellecchia et al., 2023a). De-identified data have been submitted to ClinicalTrials.gov (ID: NCT04729127) and will be available following quality control review by the National Library of Medicine.

### Setting

The parent study was conducted in publicly funded EI agencies during the COVID-19 pandemic. *Project ImPACT* sessions occurred via video conferencing for all families.

### Project ImPACT

*Project ImPACT* is an evidence-based caregiver-mediated NDBI designed to teach caregivers strategies to help their child develop social communication skills during daily routines and activities (Ingersoll & Dvortcsak, 2019; Ingersoll & Wainer, 2013). The clinician training protocol is published elsewhere (Pellecchia et al., 2023a).

### Participants

Participants included community EI clinicians and caregivers and children from the clinicians’ caseloads. Upon enrollment, children were less than 36 months of age and were either at high likelihood of or diagnosed with autism. *Project ImPACT* was delivered in English. All caregivers spoke English, and some families also spoke other languages fluently at home.

### Measures and procedures

#### Clinician and family dyad characteristics measures

Participants completed demographic questionnaires. Age, education, racial/ethnic identity, household annual income, and language were provided categorically. Household size was reported as a continuous variable.

#### Outcome measures

Trained raters evaluated clinicians’ use of coaching strategies during sessions using two measures: *Project ImPACT Coaching Fidelity Checklist* (*ImPACT Coaching Fidelity*; Ingersoll & Dvortcsak, 2019) and the *Parent Empowerment and Coaching in Early Intervention Caregiver Coaching Fidelity Tool* (*PEACE Fidelity;* Pellecchia et al., 2020).

*ImPACT Coaching Fidelity* evaluates clinician adherence to *Project ImPACT* coaching procedures (Ingersoll & Dvortcsak, 2019). A total of 18 items are scored as 0.0 (not observed), 0.5 (partially observed but not to fidelity), or 1.0 (sufficient quality); items may also be scored as *not applicable*. We created weighted fidelity scores by multiplying scores for each item by a weight based on the intervention developer’s recommendations about the item’s relevance to high-quality coaching with *Project ImPACT* (e.g. providing positive coaching comments has a weight of 3, while setting an agenda has a weight of 1; for more details see Supplemental Table S1). *ImPACT Coaching Fidelity* overall score was the sum of the weighted fidelity scores, divided by total possible points, and multiplied by 100. The total maximum possible score depends on the specific items scored as applicable for a given session.

*PEACE Fidelity* is an intervention-agnostic measure of quality and use of caregiver coaching strategies in CMI (Pellecchia et al., 2023b; Supplemental Appendix S1). The *PEACE Fidelity* includes five subcomponents: collaboration, practicing in daily routines, demonstration, practice with feedback, and reflection with problem-solving. Its 25 items are scored across subcomponents on a 5-point scale, ranging from 1 (never observed) to 5 (almost always observed). The mean of all items scored is the *PEACE Fidelity* overall fidelity score (Pellecchia et al., 2020).

#### Video recording procedures

Fidelity for both measures was scored by trained research staff. Twenty percent of videos was double-coded. Mean inter-rater agreement was 87% for *PEACE Fidelity* and 94% for *ImPACT Coaching Fidelity*. Video recordings were collected after 8–12 weeks of Project ImPACT implementation.

### Data analysis

Analyses were conducted using R version 4.1.2 (R Core Team, 2021). We examined family characteristics using descriptive statistics. Families’ reported racial and ethnic identities were categorized into one of two groups: “racially/ethnically minoritized families” or “White, non-Hispanic families.” Household language was aggregated into two groups: “exclusively English-speaking household” or “other languages household,” meaning a non-English language was spoken in the home.

We calculated descriptive statistics for both fidelity measures. Correlations between mean total scores and subcomponents of fidelity measures were computed as a basic assessment of construct convergence between the two measures (Campbell & Fiske, 1959; Strauss & Smith, 2009). Correlations between family characteristics and total scores were computed (e.g. Pearson *r* for continuous variables, point-biserial correlation *r_Pbis_* for continuous-dichotomous variables; Shmueli, 2010). Multiple linear regressions models were computed for each measure’s overall fidelity score as functions of each family characteristic variable with covariates controlling for total hours in session and parent study group assignment (i.e. families were randomized into *Project ImPACT* for 1 or 4 h per week; see Pellecchia et al., 2023a). Given the exploratory nature of this study, correlations and m﻿ultiple linear regression models were computed to assess preliminary associations (Shmueli, 2010).

We conducted analyses between family characteristics and *PEACE Fidelity* subcomponents. We estimated multiple linear regression models for each *PEACE Fidelity* subcomponent. We conducted *PEACE Fidelity* subcomponent analyses for family characteristics with statistically significant associations with overall scores. A subcomponent analysis for the *ImPACT Coaching Fidelity* was not appropriate because of parent study group assignment (Pellecchia et al., 2023a; Supplemental Appendix S2, Table S2).

## Results

### Clinician and family dyad characteristics

Table 1 presents clinician and family descriptive information.

**Table 1.** Clinician and family characteristics.

	Clinicians (*n* = 12)		Family dyad (*n* = 34)	
	*n*	%	*n*/M	%/SD
Education				
Less than high school	–	–	3	8.82
High school	–	–	8	23.53
Some college	–	–	14	41.18
Bachelor’s degree	2	16.67	8	23.53
Graduate degree	10	83.33	–	–
Unknown	–	–	1	2.94
Certification				
Teaching	7	58.33	–	–
Speech	1	8.33	–	–
BCBA/BCaBA	1	8.33	–	–
Other^a^	3	25.00	–	–
None	0	–	–	–
Race/ethnicity				
Am.Indian/Alaska Nat.	0	–	1	2.94
Asian/NHPI	0	–	2	5.88
Black	5	41.67	18	52.94
White Non-Hispanic	7	58.33	9	26.47
Latinx/Hispanic	0	–	6	17.65
Session caregiver				
Mother	–	–	29	85.29
Father	–	–	1	2.94
Grandparent	–	–	3	8.82
Legal guardian	–	–	1	2.94
Household income				
Under $20K	–	–	18	52.94
$20K–$40K	–	–	7	20.59
$40K–$60K	–	–	3	8.82
Over $60K	–	–	6	17.65
Household language				
English only	–	–	26	76.47
Other language(s) ^b^	–	–	8	23.53
House size	–	–	4.12	1.19

Race/ethnicity reporting is non-exclusive, meaning summation of percentages maybe greater than 100%. Am.Indian/Alaska Nat. = American Indian or Alaska Native; Asian/NHPI = Asian or Native Hawaiian and Pacific Islander.

a Certification Other includes Behavior Specialist, Applied Behavior Analysis.

b Other Languages include Arabic, Cambodian, Haitian Creole, Laos, Russian, Spanish.

#### Clinicians

Twelve clinicians participated. Most had a graduate degree (*n* = 10, 83.3%) and were between ages 36 and 40 (*n* = 4, 33.3%). Five clinicians were Black (41.7%) and seven were White (58.3%).

#### Family dyad

We enrolled 34 family dyads, an average of two families (range = 2–4) from each clinicians’ caseload. Children were less than 36 months of age (mean age 23.8 months; SD = 3.4); most children were male (*n* = 22, 64.7%). Participating caregivers were predominately mothers (*n* = 29, 85.29%). Most families were Black (*n* = 18, 52.9%). Eight families (23.5%) reported speaking languages other than English in their homes. Most families reported an annual household income under $20K (*n* = 18, 52.9%).

### Outcome measures

On average, coaching fidelity was low (*ImPACT Coaching Fidelity* score M = 56.9%, SD = 22.0; *PEACE Fidelity* overall score M = 2.6, SD = 0.6; Supplemental Table S3). Correlations between overall scores and fidelity subcomponents were generally moderate to high (Supplemental Table S4). *ImPACT Coaching Fidelity* and *PEACE Fidelity* overall scores were moderately correlated (*r* = 0.64, *p* < 0.01). *ImPACT Coaching Fidelity* overall score was moderately correlated with *PEACE Fidelity* in-vivo feedback and reflection & problem-solving (*r* *=* 0.56, *p* *<* 0.01). See Supplemental Table S4.

#### ImPACT coaching fidelity and family characteristics

Correlations between *ImPACT Coaching Fidelity* score and family education, minoritized racial/ethnic status, household income, and household size were small and not statistically significant. The correlation between *ImPACT Coaching Fidelity* score and household language was low and statistically significant (*r_Pbis_* = 0.34, *p* = 0.05). Visual inspection demonstrated a curvilinear relationship between *ImPACT Coaching Fidelity* score and household income.

**Figure 1.**
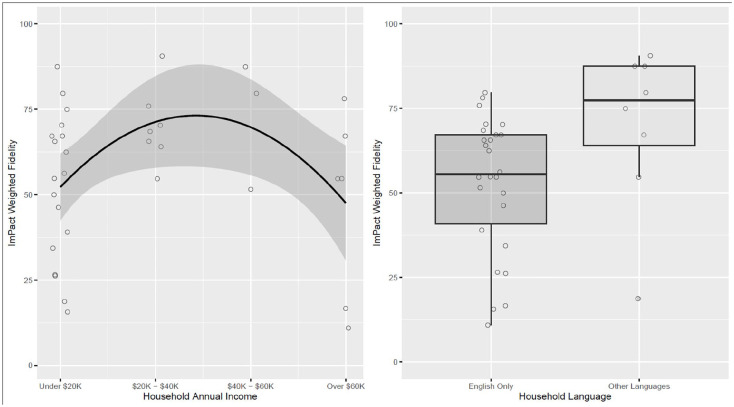
ImPACT coaching fidelity overall score × family characteristics. Figure displays significant associations (*p* > 0.05) between family characteristics and *ImPACT Coaching Fidelity* overall score (ImPACT Weighted Fidelity). ^a^ Household annual income demonstrated a significant curvilinear association with *ImPACT coaching fidelity* overall score with income at the lowest and highest ends having lower and more variable *ImPACT Coaching Fidelity* overall score. ^b^ Household language demonstrated a significant association with *ImPACT Coaching Fidelity* overall score where households speaking a language other than English are associated with higher *ImPACT Coaching Fidelity* overall scores.

Multivariate models did not demonstrate significant associations between clinicians’ adherence to *Project ImPACT* coaching and caregiver education, family racial/ethnic identity, or household size (Table 2). Household income and language were significantly associated with *ImPACT Coaching Fidelity* overall score (Table 2; Figure 1(a) and (b)). A multiple linear regression was estimated with household income as a quadratic term to better fit this relationship between household income and coaching fidelity. The association between household income and *ImPACT Coaching Fidelity* overall score was statistically significant, *b* = −10.4, standard error (SE) = 4.1, *p* < 0.05. The quadratic term for household income accounted for an additional 18% of the variance in coaching fidelity. Coaching fidelity was lower and more variable for households with income at or below $20K and above $60K compared to families with household incomes between $20K and $40K and between $40K and $60K. The association between household language and *ImPACT Coaching Fidelity* score was statistically significant, *b* = 18.5, *SE* = 8.8, *p* < 0.05; clinicians’ *Project ImPACT* coaching adherence was lower when coaching caregivers in households where English was the only language spoken.

**Table 2.** Fidelity overall scores × family characteristics multivariate linear regression results.

Model	ImPACT		PEACE	
	*b*	95% CI [LL, UL]	*b*	95% CI [LL, UL]
Caregiver education				
Intercept	60.52**	[27.34, 93.70]	2.99**	[2.17, 3.80]
High school	−4.28	[−35.45, 26.89]	0.13	[−0.63, 0.90]
Some college	−7.09	[−36.48, 22.30]	−0.24	[−0.96, 0.48]
>Bachelor’s degree	1.12	[−31.36, 33.61]	−0.30	[−1.09, 0.50]
Total EI hours	−0.11	[−0.64, 0.42]	−0.01	[−0.02, 0.01]
ImPACT 4 h/week	13.06	[−16.44, 42.56]	0.30	[−0.42, 1.02]
Racial/ethnic identity				
Intercept	47.44**	[26.74, 68.13]	2.70**	[2.16, 3.24]
Racial/ethnic minority	9.91	[−10.87, 30.69]	−0.05	[−0.59, 0.50]
Total EI hours	−0.04	[−0.56, 0.49]	−0.00	[−0.01, 0.01]
ImPACT 4 h/week	6.41	[−22.58, 35.41]	0.00	[−0.76, 0.77]
Household income				
Intercept	10.82	[−28.64, 50.28]	1.88**	[0.80, 2.95]
Household income	50.78*	[9.27, 92.28]	0.94	[−0.19, 2.07]
Household income^2^	−10.39*	[−18.87, −1.92]	−0.19	[−0.42, 0.04]
Total EI hours	−0.05	[−0.52, 0.42]	−0.00	[−0.02, 0.01]
ImPACT 4 h/week	6.11	[−20.36, 32.57]	0.10	[−0.62, 0.82]
Household language				
Intercept	47.92**	[31.83, 64.01]	2.62**	[2.18, 3.06]
Household non-English lang.	18.45*	[0.54, 36.35]	0.16	[−0.33, 0.65]
Total EI hours	0.06	[−0.40, 0.53]	−0.00	[−0.01, 0.01]
ImPACT 4 h/week	3.49	[−22.70, 29.67]	0.04	[−0.68, 0.76]
Household size				
Intercept	41.89*	[8.56, 75.22]	2.92**	[2.06, 3.79]
Household size	3.01	[−4.05, 10.08]	−0.07	[−0.25, 0.12]
Total EI hours	0.09	[−0.42, 0.60]	−0.00	[−0.02, 0.01]
ImPACT 4 h/week	−2.06	[−30.99, 26.87]	0.13	[−0.62, 0.88]

LL and UL indicate the lower and upper limits of a confidence interval, respectively.

* *p* < 0.05; **p < 0.01.

#### PEACE Fidelity and family characteristics

##### PEACE Fidelity overall score

Correlations between *PEACE Fidelity* score and family characteristics were not statistically significant. *PEACE Fidelity* score was not associated with any family characteristics (Table 3).

**Table 3.** PEACE Fidelity subcomponents × family characteristics multivariate linear regression results.

Models	PEACE collaboration		PEACE daily routines		PEACE demonstration		PEACE invivo feedback		PEACE reflection and problem-solving	
	*b*	95% CI	*b*	95% CI	*b*	95% CI	*b*	95% CI	*b*	95% CI
		[LL, UL]		[LL, UL]		[LL, UL]		[LL, UL]		[LL, UL]
Household income										
Intercept	1.96*	[0.43, 3.48]	1.51**	[0.62, 2.40]	2.37*	[0.30, 4.45]	1.22	[−1.03, 3.47]	2.29**	[1.08, 3.50]
Household income	0.64	[−0.97, 2.24]	−0.15	[−1.08, 0.79]	0.95	[−1.23, 3.13]	2.43*	[0.06, 4.80]	1.04	[−0.24, 2.31]
Household income^2^	−0.16	[−0.48, 0.17]	0.06	[−0.14, 0.25]	−0.21	[−0.66, 0.24]	−0.49*	[−0.97, −0.01]	−0.21	[−0.47, 0.05]
Total EI hours	−0.01	[−0.02, 0.01]	0.00	[−0.01, 0.02]	0.00	[−0.03, 0.02]	−0.02	[−0.04, 0.01]	0.00	[−0.01, 0.01]
ImPACT 4 h/week	0.32	[−0.70, 1.34]	−0.30	[−0.90, 0.29]	−0.18	[−1.57, 1.22]	1.28^†^	[−0.23, 2.78]	−0.26	[−1.07, 0.55]
Household language										
Intercept	2.41**	[1.79, 3.03]	1.57**	[1.20, 1.93]	2.95**	[2.13, 3.78]	3.03**	[2.13, 3.94]	3.18**	[2.68, 3.68]
Household non-English lang.	−0.16	[−0.85, 0.52]	−0.12	[−0.53, 0.29]	0.44	[−0.47, 1.36]	0.92	[−0.09, 1.93]	0.01	[−0.54, 0.57]
Total EI hours	−0.01	[−0.02, 0.01]	0.01	[−0.01, 0.02]	0.00	[−0.03, 0.02]	−0.01	[−0.04, 0.02]	0.00	[−0.01, 0.02]
ImPACT 4 h/week	0.34	[−0.67, 1.35]	−0.38	[−0.98, 0.22]	−0.17	[−1.51, 1.17]	1.13	[−0.35, 2.60]	−0.35	[−1.17, 0.46]

*LL* and *UL* indicate the lower and upper limits of a confidence interval, respectively.

† *p* < 0.10; *p < 0.05; **p < 0.01.

##### PEACE Fidelity subcomponent scores

**Figure 2.**
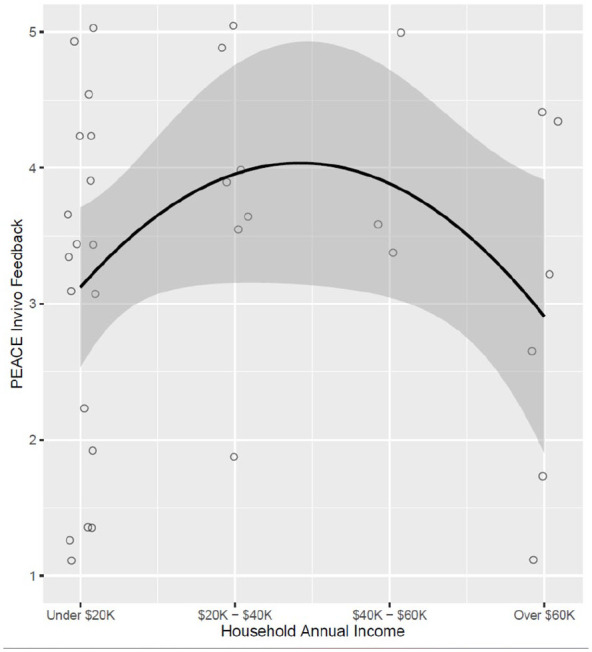
PEACE Fidelity in vivo feedback × household annual income. Figure displays significant associations (*p* > 0.05) between household annual income and *PEACE Fidelity* in vivo feedback score. Household annual income demonstrated a significant curvilinear association with *PEACE Fidelity* in vivo feedback score with income at the lowest and highest ends having lower *PEACE Fidelity* in vivo feedback scores.

Subcomponent-specific models were run per each *PEACE Fidelity* subcomponent with household income and language (Table 3). The association between families’ household income and clinicians’ *PEACE Fidelity* in-vivo feedback was statistically significant, *b* = −0.5, *SE* = 0.2, *p* < 0.05, and demonstrated a curvilinear relationship (Figure 2). Neither household income nor language was associated with any other *PEACE Fidelity* subcomponents.

For transparency and robustness check, significant results were computed without covariates (Supplemental Table S5; Simmons et al., 2011). Household income remained significantly associated with *ImPACT Coaching Fidelity* overall score, *b* = −10.3, *SE* = 4.1, *p* < 0.05. Other results were no longer statistically significant (*p* < 0.07).

## Discussion

We found associations between household income and language with clinicians’ coaching fidelity. Families at the lowest and highest ends of the income range received less *Project ImPACT* coaching manual adherence than middle-income families, suggesting a more nuanced relationship than previously appreciated. A significant curvilinear relationship was similarly observed with *PEACE Fidelity* in vivo feedback and income. These results align with qualitative studies where clinicians report that challenges such as housing instability, food insecurity, and lack of play materials or other essential resources create barriers to coaching lower-income families (Straiton et al., 2021; Tomczuk et al., 2022). Low coaching fidelity among higher-income families may reflect clinicians perceiving reduced authority or a preference among these caregivers for clinician-directed sessions focused on their child, rather than caregiver-focused coaching.

*ImPACT Coaching Fidelity* overall scores were higher for families that spoke languages other than English. These results may stem from language differences between clinicians and caregivers that reduce casual conversation, allowing more time for coaching. While prior research highlights barriers such as challenges engaging caregivers and discomfort providing feedback to culturally and linguistically diverse (CLD) families (Stewart & Applequist, 2019), our findings present mixed results. This suggests a need for more expansive and mechanistic studies to better understand these associations.

*ImPACT Coaching Fidelity* and *PEACE Fidelity* overall scores and subcomponent scores were moderately and positively correlated (Supplemental Table S4), yet fidelity scores were differentially associated with family characteristics. In vivo feedback had both the highest mean score and the greatest variability (M = 3.32, SD = 1.26) among *PEACE Fidelity* subcomponents (Supplemental Table S4). It may be that the subcomponent-level *PEACE Fidelity* in vivo feedback presents with greater signal relative to other subcomponents (e.g. daily routines; M = 1.58, SD = 0.48). Differences in coaching fidelity at the subcomponent level are consistent with differences in intention to use different coaching components (Pellecchia et al., 2024).

This study is an exploratory analysis with a small sample size. Therefore, results should be interpreted with caution. Family characteristic variables were not selected a priori, limiting, for example, income ranges. Yet, household income association with coaching fidelity demonstrated consistency across *ImPACT Coaching Fidelity* score and *PEACE Fidelity* in vivo feedback.

## Conclusion

We provide preliminary evidence that family characteristics and clinicians’ coaching fidelity are associated. This study responds to calls for more inclusive autism research by engaging and enrolling diverse, marginalized families, with the goal of advancing health equity (Machalicek et al., 2022; Maye et al., 2022; Straiton et al., 2024). Future research should collect more comprehensive family characteristic data. This study sets the stage for research to ameliorate and improve associations between family characteristics, circumstances, and clinicians’ caregiver coaching.

## Supplemental Material

sj-docx-1-aut-10.1177_13623613251317780 – Supplemental material for Short report: Associations of family characteristics and clinicians’ use of caregiver coaching in early intervention Supplemental material, sj-docx-1-aut-10.1177_13623613251317780 for Short report: Associations of family characteristics and clinicians’ use of caregiver coaching in early intervention by Alyssa M Hernandez, Diondra Straiton, David S Mandell, Brooke Ingersoll, Samantha Crabbe, Sarah Rieth and Melanie Pellecchia in Autism

## References

[bibr1-13623613251317780] Aranbarri A. Stahmer A. C. Talbott M. R. Miller M. E. Drahota A. Pellecchia M. Barber A. B. Griffith E. M. Morgan E. H. Rogers S. J. (2021). Examining US public early intervention for toddlers with autism: Characterizing services and readiness for evidence-based practice implementation. Frontiers in Psychiatry, 12, Article 786138. 10.3389/fpsyt.2021.786138 PMC8716593 34975582

[bibr2-13623613251317780] Begeer S. Bouk S. E. Boussaid W. Terwogt M. M. Koot H. M. (2009). Underdiagnosis and referral bias of autism in ethnic minorities. Journal of Autism and Developmental Disorders, 39(1), 142–148. 10.1007/s10803-008-0611-5 18600440

[bibr3-13623613251317780] Brookman-Frazee L. I. Drahota A. Stadnick N. (2012). Training community mental health therapists to deliver a package of evidence-based practice strategies for school-age children with autism spectrum disorders: A pilot study. Journal of Autism and Developmental Disorders, 42(8), 1651–1661. 10.1007/s10803-011-1406-7 22102293 PMC3546546

[bibr4-13623613251317780] Campbell D. T. Fiske D. W. (1959). Convergent and discriminant validation by the multitraitmultimethod matrix. Psychological Bulletin, 56(2), 81–105. 13634291

[bibr5-13623613251317780] Carr T. Shih W. Lawton K. Lord C. King B. Kasari C. (2016). The relationship between treatment attendance, adherence, and outcome in a caregiver-mediated intervention for low-resourced families of young children with autism spectrum disorder. Autism, 20(6), 643–652. 10.1177/1362361315598634 26290524

[bibr6-13623613251317780] Cheng W. M. Smith T. B. Butler M. Taylor T. M. Clayton D. (2023). Effects of parent-implemented interventions on outcomes of children with autism: A meta-analysis. Journal of Autism and Developmental Disorders, 53(11), 4147–4163. 10.1007/s10803-022-05688-8 35996037 PMC10539413

[bibr7-13623613251317780] Fountain C. Winter A. S. Bearman P. S. (2012). Six developmental trajectories characterize children with autism. Pediatrics, 129(5), e1112–e1120. 10.1542/peds.2011-1601 PMC3340586 22473372

[bibr8-13623613251317780] Fuller E. A. Kaiser A. P. (2020). The effects of early intervention on social communication outcomes for children with autism spectrum disorder: A meta-analysis. Journal of Autism and Developmental Disorders, 50(5), 1683–1700. 10.1007/s10803-019-03927-z 30805766 PMC7350882

[bibr9-13623613251317780] Green J. Leadbitter K. Ellis C. Taylor L. Moore H. L. Carruthers S. James K. Taylor C. Balabanovska M. Langhorne S. Aldred C. Slonims V. Grahame V. Parr J. Humphrey N. Howlin P. McConachie H. Couteur A. L. Charman T. Pickles A. (2022). Combined social communication therapy at home and in education for young autistic children in England (PACT-G): A parallel, single-blind, randomised controlled trial. The Lancet Psychiatry, 9(4), 307–320. 10.1016/S2215-0366(22)00029-3 35305746 PMC9630149

[bibr10-13623613251317780] Hume K. Steinbrenner J. R. Odom S. L. Morin K. L. Nowell S. W. Tomaszewski B. Szendrey S. McIntyre N. S. Yücesoy-Özkan S. Savage M. N. (2021). Evidence-based practices for children, youth, and young adults with autism: Third generation review. Journal of Autism and Developmental Disorders, 51(11), 4013–4032. 10.1007/s10803-020-04844-2 33449225 PMC8510990

[bibr11-13623613251317780] Individuals With Disabilities Education Act, 20 U.S.C. § 1400. (2004). http://uscode.house.gov/view.xhtml?path=/prelim@title20/chapter33/subchapter3&edition=prelim

[bibr12-13623613251317780] Ingersoll B. Dvortcsak A. (2019). Teaching social communication to children with autism and other developmental delays: The project ImPACT guide to coaching parents and the project ImPACT manual for parents (2 book set). Guilford Press.

[bibr13-13623613251317780] Ingersoll B. Wainer A. (2013). Initial efficacy of project ImPACT: A parent-mediated social communication intervention for young children with ASD. Journal of Autism and Developmental Disorders, 43(12), 2943–2952. 10.1007/s10803-013-1840-9 23689760

[bibr14-13623613251317780] Lawson G. M. Mandell D. S. Tomczuk L. Fishman J. Marcus S. C. Pellecchia M. (2022). Clinician intentions to use the components of parent coaching within community early intervention systems. Administration and Policy in Mental Health and Mental Health Services Research, 50, 357–365. 10.1007/s10488-022-01243-w 36525093 PMC10191901

[bibr15-13623613251317780] Liu B. M. Paskov K. Kent J. McNealis M. Sutaria S. Dods O. Harjadi C. Stockham N. Ostrovsky A. Wall D. P. (2023). Racial and Ethnic Disparities in Geographic Access to Autism Resources Across the US. JAMA Network Open, 6(1), e2251182. 10.1001/jamanetworkopen.2022.51182 PMC9871799 36689227

[bibr16-13623613251317780] Machalicek W. Glugatch L. Erturk B. Brafford T. Kunze M. Drew C. Douglas A. Storie S. Crowe R. Magaña S. (2022). Recommendations for diversifying racial and ethnic representation in autism intervention research: A crossover review of recruitment and retention practices in pediatric mental health. Journal of Clinical Medicine, 11(21), Article 6468. 10.3390/jcm11216468 PMC9654487 36362698

[bibr17-13623613251317780] Magaña S. Lopez K. Aguinaga A. Morton H. (2013). Access to diagnosis and treatment services among Latino children with autism spectrum disorders. Intellectual and Developmental Disabilities, 51(3), 141–153. 10.1352/1934-9556-51.3.141 23834211

[bibr18-13623613251317780] Magaña S. Parish S. L. Rose R. A. Timberlake M. Swaine J. G. (2012). Racial and ethnic disparities in quality of health care among children with autism and other developmental disabilities. Intellectual and Developmental Disabilities, 50(4), 287–299. 10.1352/1934-9556-50.4.287 22861130

[bibr19-13623613251317780] Maye M. Boyd B. A. Martínez-Pedraza F. Halladay A. Thurm A. Mandell D. S. (2022). Biases, barriers, and possible solutions: Steps towards addressing autism researchers under-engagement with racially, ethnically, and socioeconomically diverse communities. Journal of Autism and Developmental Disorders, 52(9), 4206–4211. 10.1007/s10803-021-05250-y 34529251 PMC8924013

[bibr20-13623613251317780] Pellecchia M. Beidas R. S. Mandell D. S. Cannuscio C. C. Dunst C. J. Stahmer A. C. (2020). Parent empowerment and coaching in early intervention: Study protocol for a feasibility study. Pilot and Feasibility Studies, 6(1), Article 22. 10.1186/s40814-020-00568-3 PMC7020349 32082608

[bibr21-13623613251317780] Pellecchia M. Ingersoll B. Marcus S. C. Rump K. Xie M. Newman J. Zeigler L. Crabbe S. Straiton D. Chávez E. C. Mandell D. S. (2023a). Pilot randomized trial of a caregiver-mediated naturalistic developmental behavioral intervention in part C early intervention. Journal of Early Intervention, 46, 194–216. 10.1177/10538151231217462

[bibr22-13623613251317780] Pellecchia M. Mandell D. S. Beidas R. S. Dunst C. J. Tomczuk L. Newman J. Zeigler L. Stahmer A. C. (2022). Parent coaching in early intervention for autism spectrum disorder: A brief report. Journal of Early Intervention, 45, 185–197. 10.1177/10538151221095860 37655268 PMC10469633

[bibr23-13623613251317780] Pellecchia M. Mandell D. S. Beidas R. S. Dunst C. J. Tomczuk L. Newman J. Zeigler L. Stahmer A. C. (2023b). Parent coaching in early intervention for autism spectrum disorder: A brief report. Journal of Early Intervention, 45(2), 185–197. 10.1177/10538151221095860 37655268 PMC10469633

[bibr24-13623613251317780] Pellecchia M. Mandell D. S. Tomczuk L. Marcus S. C. Stewart R. Stahmer A. C. Beidas R. S. Rieth S. R. Lawson G. M. (2024). A mixed-methods evaluation of organization and individual factors influencing provider intentions to use caregiver coaching in community-based early intervention. Implementation Science Communications, 5(1), Article 17. 10.1186/s43058-024-00552-5 PMC10900730 38414019

[bibr25-13623613251317780] R Core Team. (2021). R: A language and environment for statistical computing [Computer software]. R Foundation for Statistical Computing. https://www.R-project.org/

[bibr26-13623613251317780] Reichow B. Wolery M. (2009). Comprehensive synthesis of early intensive behavioral interventions for young children with autism based on the UCLA young autism project model. Journal of Autism and Developmental Disorders, 39(1), 23–41. 10.1007/s10803-008-0596-0 18535894

[bibr27-13623613251317780] Shmueli G. (2010). To explain or to predict? Statistical Science, 25(3), 289–310. 10.1214/10-STS330

[bibr28-13623613251317780] Simmons J. P. Nelson L. D. Simonsohn U. (2011). False-positive psychology: Undisclosed flexibility in data collection and analysis allows presenting anything as significant. Psychological Science, 22(11), 1359–1366. 10.1177/0956797611417632 22006061

[bibr29-13623613251317780] Stewart S. L. Applequist K. (2019). Diverse families in early intervention: Professionals’ views of coaching. Journal of Research in Childhood Education, 33(2), 242–256. 10.1080/02568543.2019.1577777

[bibr30-13623613251317780] Straiton D. Groom B. Ingersoll B. (2021). A mixed methods exploration of community providers’ perceived barriers and facilitators to the use of parent training with Medicaid-enrolled clients with autism. Autism, 25(5), 1368–1381. 10.1177/1362361321989911 33557590

[bibr31-13623613251317780] Straiton D. Pomales-Ramos A. Broder-Fingert S. (2024). A health equity perspective on autism diagnosis: Progress and future research priorities. Pediatrics, 154(4), Article e2023064262. 10.1542/peds.2023-064262 PMC11422189 39233661

[bibr32-13623613251317780] Strauss M. E. Smith G. T. (2009). Construct validity: Advances in theory and methodology. Annual Review of Clinical Psychology, 5(1), 1–25. 10.1146/annurev.clinpsy.032408.153639 PMC2739261 19086835

[bibr33-13623613251317780] Tomczuk L. Stewart R. E. Beidas R. S. Mandell D. S. Pellecchia M. (2022). Who gets coached? A qualitative inquiry into community clinicians’ decisions to use care giver coaching. Autism, 26(3), 575–585. 10.1177/13623613211059499 34866429 PMC8934260

[bibr34-13623613251317780] Zwaigenbaum L. Bauman M. L. Choueiri R. Kasari C. Carter A. Granpeesheh D. Mailloux Z. Smith Roley S. Wagner S. Fein D. Pierce K. Buie T. Davis P. A. Newschaffer C. Robins D. Wetherby A. Stone W. L. Yirmiya N. Estes A. Natowicz M. R. (2015). Early intervention for children with autism spectrum disorder under 3 years of age: Recommendations for practice and research. Pediatrics, 136(Supplement_1), S60–S81. 10.1542/peds.2014-3667E PMC9923898 26430170

