# Supplementary material for: Short report: Associations of family characteristics and clinicians’ use of caregiver coaching in early intervention
Source: Autism. 2025 Feb 11;29(7):1898–906. doi: 10.1177/13623613251317780 (PMC12159352; doi:10.1177/13623613251317780)
Supplement: sj-docx-1-aut-10.1177_13623613251317780 – Supplemental material for Short report: Associations of family characteristics and clinicians’ use of caregiver coaching in early intervention [file sj-docx-1-aut-10.1177_13623613251317780.docx]

**Supplementary Material**

[MASKED].

**Short Report: Associations of Family Characteristics and Clinicians’ Use of Caregiver Coaching in Early Intervention**

**Table S1. *ImPACT Coaching Fidelity* Item Weighting**

**Appendix S1.** *PEACE Caregiver Coaching Fidelity Tool* measure

**Appendix S2.** Description of primary study constraints and NAs

**Table S2.** NAs across group assignment for fidelity measures

**Table S3.** Descriptive statistics of fidelity measures

**Table S4.** *ImPACT Coaching Fidelity* and *PEACE Fidelity* overall score and subcomponent-level correlations

**Table S5.** Fidelity scores x family characteristics multivariate linear regression results without covariates

**Table S1.** *ImPACT Coaching Fidelity Item Weighting*

| **Item** | **Prepare for the session** | **Weight** |
| --- | --- | --- |
|  | **Check in and set the session agenda** |  |
| 1 | The coach greets the family warmly, sets the agenda, and addresses concerns as appropriate. | 1 |
|  | **Review the Practice Plan** |  |
| 2 | The coach reviews the written Practice Plan from the previous session with the parent. | 2 |
| 3 | The coach helps the parent problem-solve any issues from the previous week. | 2 |
|  | **Introduce the new technique(s)** |  |
| 4 | The coach introduces the technique(s), explains the rationale, and discusses key elements. | 2 |
| 5 | The coach asks questions to assess the parent’s understanding of the information. | 1 |
|  | **Demonstrate the technique(s)** |  |
| 6 | The coach prepares for the demonstration by asking the parent to watch for the technique and its impact on the child. | 1 |
| 7 | shows video examples/ The coach demonstrates the technique(s) with the child, while describing what she is doing and explaining its impact on the child’s behavior. | 2 |
| 8 | The coach asks the parent to reflect on what she observed. | 1 |
|  | **Have the parent practice, and give feedback** |  |
| 9 | The coach encourages the parent to practice technique(s) with the child. | 3 |
| 10 | The coach provides effective positive feedback to the parent. | 3 |
| 11 | The coach provides effective corrective feedback to the parent. | 3 |
|  | **Help the parent reflect, and plan for practice** |  |
| 12 | The coach helps the parent reflect on the in-session practice. | 1 |
| 13 | The coach helps the parent select goals and activities and complete the sequence graphic on the Practice Plan. | 2 |
| 14 | The coach helps the parent problem-solve potential challenges. | 2 |
| 15 | The coach assigns reflection and reading for the following session. | 1 |
|  | **Use appropriate coaching style** |  |
| 16 | The coach uses a collaborative interaction style throughout the session. | 1 |
| 17 | The coach is responsive to the family’s beliefs, values, and culture. | 1 |
| 18 | The coach uses a strengths-based approach. | 3 |
|  | Total possible points | 32 |

**Appendix S1.** *PEACE Caregiver Coaching Fidelity Tool* measure

**Appendix S1.** *PEACE Caregiver Coaching Fidelity Tool* measure

**RATER: _________________** **DATE: _________________ Participant ID: _____________________**

| **Parent Coaching Fidelity Form – TELEHEALTH VERSION** | | | | | | | |
| --- | --- | --- | --- | --- | --- | --- | --- |
| ***When there was an opportunity, did the provider*** | ***Almost Always*** | ***Often*** | ***Sometimes*** | ***Rarely*** | ***Never*** | ***No Opportunity*** | ***Comments*** |
| **General** |  |  |  |  |  |  |  |
| 1. Work through any technology issues during session with parent (adjust camera position, etc). | 5 | 4 | 3 | 2 | 1 | N |  |
| 1. Create/maintain opportunities for caregiver and child to interact. | 5 | 4 | 3 | 2 | 1 |  |  |
| 1. The coach skillfully balances child attention and parent explanation/description. | 5 | 4 | 3 | 2 | 1 |  |  |
| **Collaboration** |  |  |  |  |  |  | Mean score: |
| 1. Let caregivers make decisions and take charge of the intervention session. | 5 | 4 | 3 | 2 | 1 |  |  |
| 1. Use and expand caregiver ideas during a session. | 5 | 4 | 3 | 2 | 1 |  |  |
| 1. The coach and parent collaboratively set goals for child’s progress. | 5 | 4 | 3 | 2 | 1 |  |  |
| 1. Ask for caregiver input or invite feedback on what is observed. | 5 | 4 | 3 | 2 | 1 |  |  |
| **Daily Routines** |  |  |  |  |  |  | Mean score: |
| 1. Engage caregiver and child in activities that are related to their usual daily routines. | 5 | 4 | 3 | 2 | 1 |  |  |
| 1. Explain how embedding strategies in daily routines helps child development. | 5 | 4 | 3 | 2 | 1 |  |  |
| 1. Connect skills being learned in current routines to other/future routines. | 5 | 4 | 3 | 2 | 1 |  |  |
| 1. Suggest things to do with the child within and outside the intervention session. | 5 | 4 | 3 | 2 | 1 |  |  |

| ***When there was an opportunity, did the provider*** | ***Almost Always*** | ***Often*** | ***Sometimes*** | ***Rarely*** | ***Never*** | ***No Opportunity*** | ***Comments*** |
| --- | --- | --- | --- | --- | --- | --- | --- |
| **Demonstration** |  |  |  |  |  |  | Mean score: |
| 1. Explicitly teach a strategy to the caregiver. | 5 | 4 | 3 | 2 | 1 |  |  |
| 1. Explain the purpose of techniques implemented. | 5 | 4 | 3 | 2 | 1 |  |  |
| 1. Demonstrate techniques that promote parent-child interaction – *via video clips or modeling of verbal strategies*. | 5 | 4 | 3 | 2 | 1 |  |  |
| **In-Vivo Feedback** |  |  |  |  |  |  | Mean score: |
| 1. Comment on specific strategies that are working well (*positive* feedback). | 5 | 4 | 3 | 2 | 1 |  |  |
| 1. Observe ongoing interactions and provide (*constructive*) feedback about current actions. | 5 | 4 | 3 | 2 | 1 |  |  |
| 1. Allow sufficient time for the caregiver to practice strategies. | 5 | 4 | 3 | 2 | 1 |  |  |
| **Reflection and Problem**  **Solving** |  |  |  |  |  |  | Mean score: |
| 1. Answer caregiver concerns. | 5 | 4 | 3 | 2 | 1 | N |  |
| 1. Listen to what the caregiver has to say. | 5 | 4 | 3 | 2 | 1 | N |  |
| 1. Evaluate progress with the caregiver. | 5 | 4 | 3 | 2 | 1 |  |  |
| 1. Ask caregiver questions about routines, use of strategies, or child’s actions. | 5 | 4 | 3 | 2 | 1 |  |  |
| 1. The coach helps the parent work through any obstacles in the implementation of the techniques using reflective strategies. | 5 | 4 | 3 | 2 | 1 | N |  |
| 1. The coach asks the parent about possible barriers to practice and discusses solutions. | 5 | 4 | 3 | 2 | 1 |  |  |
| **Overall Fidelity Score (Mean of all items scored):** | | | | | | | |

**Appendix S2.** Description of primary study constraints and NAs

The primary study (Pellecchia et al., 2023a) was a pilot randomized trial with participants in three conditions: (a) treatment as usual, (b) *ImPACT* for 1 hour/week (*n ­*= 17), and (c) *ImPACT* for 4 hours/week (n = 17). Conditions (b) and (c) were included in this study. In condition (b), clinicians were required to cover all *ImPACT*-specific procedures in one session per week. In condition (c) four one-hour sessions per week occurred allowing for *ImPACT*-specific procedures to occur over many sessions per week. Only one one-hour session per week was assessed for coaching fidelity regardless of condition type. Table S1 descriptively demonstrates procedural-level differences between groups. There were a higher number of NAs or *not applicable* items within *ImPACT* procedures for participants in condition (c) compared to condition (b). Note: *PEACE* assesses quality and use of caregiver coaching strategies agnostic to intervention-specific procedures, whereas ImPACT fidelity assess intervention-specific procedural adherence. Across conditions, no items were *not applicable* for *PEACE fidelity* evaluation.

**Table S2.**

*Not applicable items (NAs) across group assignment for fidelity measures*

| Group Assignment | 1 Hour  (*n =* 17) | | 4 Hour  (n = 17) | |
| --- | --- | --- | --- | --- |
|  | | NAs | | NAs |
| *ImPACT* | |  | |  |
| Review the practice plan | | 0 | | 6 |
| Introduce the new technique(s) | | 0 | | 9 |
| Demonstrate the technique(s) | | 2 | | 9 |
| Have parent practice & give feedback | | 0 | | 0 |
| Help parent reflect & plan for practice | | 0 | | 0 |
| *PEACE* | |  | |  |
| Collaboration | | 0 | | 0 |
| Daily routines | | 0 | | 0 |
| Demonstration | | 0 | | 0 |
| Invivo-feedback | | 0 | | 0 |
| Reflect & problem solving | | 0 | | 0 |

**Table S3**

*Descriptive statistics of fidelity measures*

|  | *M* | *SD* | Min | Max |
| --- | --- | --- | --- | --- |
| *ImPACT* |  |  |  |  |
| Total score | 0.57 | 0.22 | 0.14 | 0.94 |
| Review the practice plan | 0.43 | 0.40 | 0.00 | 1.00 |
| Introduce the new technique(s) | 0.52 | 0.41 | 0.00 | 1.00 |
| Demonstrate the technique(s) | 0.53 | 0.37 | 0.00 | 1.00 |
| Have the parent practice & give feedback | 0.58 | 0.36 | 0.00 | 1.00 |
| Help the parent reflect & plan for practice | 0.47 | 0.38 | 0.00 | 1.00 |
| *PEACE* |  |  |  |  |
| Total score | 2.62 | 0.57 | 1.28 | 3.70 |
| Collaboration | 2.29 | 0.80 | 1.00 | 4.00 |
| Daily routines | 1.58 | 0.48 | 1.00 | 2.50 |
| Demonstration | 2.92 | 1.07 | 1.00 | 5.00 |
| *In Vivo* Feedback | 3.32 | 1.26 | 1.00 | 5.00 |
| Reflection & Problem Solving | 3.09 | 0.65 | 1.50 | 4.33 |
| *Note*. Descriptive statistics were calculated with raw item scores | | | | |

**Table S4**

*ImPACT Coaching Fidelity and PEACE Fidelity overall scores and subcomponent-level correlations*

| Variable | 1 | 2 | 3 | 4 | 5 | 6 | 7 | 8 | 9 | 10 | 11 |
| --- | --- | --- | --- | --- | --- | --- | --- | --- | --- | --- | --- |
| 1. ImP Total |  |  |  |  |  |  |  |  |  |  |  |
| 2. PEA Total | .64** |  |  |  |  |  |  |  |  |  |  |
| 3. PEA Collaboration | .50** | .71** |  |  |  |  |  |  |  |  |  |
| 4. PEA Daily routines | -.03 | .49** | .23 |  |  |  |  |  |  |  |  |
| 5. PEA Demonstration | .36* | .77** | .39* | .43* |  |  |  |  |  |  |  |
| 6. PEA Invivo feedback | .56** | .56** | .21 | -.14 | .26 |  |  |  |  |  |  |
| 7. PEA Reflection & problem solving | .56** | .85** | .53** | .51** | .60** | .29 |  |  |  |  |  |
| 8. ImP Review the practice plan | .69** | .56** | .26 | .16 | .52** | .36 | .57** |  |  |  |  |
| 9. ImP Introduce the new technique(s) | .69** | .45* | .47* | .08 | .42* | .13 | .43* | .57** |  |  |  |
| 10. ImP Demonstrate the technique(s) | .88** | .63** | .53** | -.03 | .58** | .40 | .64** | .64** | .60** |  |  |
| 11. ImP Have the parent practice & give feedback | .77** | .51** | .32 | -.13 | .16 | .77** | .35* | .44* | .43* | .58** |  |
| 12. ImP Help the parent reflect & plan for practice | .69** | .48** | .46** | .04 | .28 | .29 | .45** | .33 | .23 | .56** | .38* |

*Note.* * indicates *p* < .05. ** indicates *p* < .01. ImP = *ImPACT*, PEA = *PEACE;* Correlations were calculated with raw item scores.

**Table S5**

*Fidelity scores x family characteristics multivariate linear regression results without covariates*

| Model | Predictor | *b* | *b*  95% CI  [LL, UL] |
| --- | --- | --- | --- |
| ***ImPACT* *Coaching* *Fidelity* Overall Score** | | | |
| Household Income | Intercept | 12.46 | [-24.77, 49.68] |
|  | Household Income | 50.08* | [10.47, 89.69] |
|  | Household Income^2^ | -10.32* | [-18.43, -2.22] |
| Household Language | Intercept | 52.80** | [44.39, 61.21] |
|  | Household NonEnglish Lang | 17.32† | [-0.02, 34.66] |
| ***PEACE Fidelity* In-vivo Feedback** | | | |
| Household Income | Intercept | 1.38 | [-0.84, 3.60] |
|  | Household Income | 2.19† | [-0.17, 4.55] |
|  | Household Income^2^ | -0.45† | [-0.94, 0.03] |

*Note.* *LL* and *UL* indicate the lower and upper limits of a confidence interval, respectively.
†p < .10. * indicates p < .05. ** indicates p < .01.
